# Supplementary material for: Heat adaptation of phage T7 under an extended genetic code
Source: Virus Evol. 2021 Dec 1;7(2):veab100. doi: 10.1093/ve/veab100 (PMC8923235; doi:10.1093/ve/veab100)
Supplement: veab100_Supp [file veab100_supp.zip › Sequencing_Analysis.zip!/Sequencing_Analysis/A1/evidence/SNP_13.html]

BRESEQ :: Evidence


| Predicted mutation | | | | | | |
| --- | --- | --- | --- | --- | --- | --- |
| evidence | seq id | position | mutation | annotation | gene | description |
| RA | NC\_001604.1 | 35,366 | C→A | intergenic (–/–) | – / – | –/– |

| Read alignment evidence... | | | | | | | | | | | |
| --- | --- | --- | --- | --- | --- | --- | --- | --- | --- | --- | --- |
|  | seq id | position | | ref | new | freq | score (cons/poly) | reads | annotation | genes | product |
| \* | NC\_001604.1 | 35,366 | 0 | C | A | 83.9% | 4137.2 / inf | 3061 | intergenic (–/–) | *–/–* | –/– |
| Reads supporting (aligned to +/- strand):  **ref** base C (313/176);  **new** base A (1438/1100);  **total** (1771/1290) | | | | | | | | | | | |
| Fisher's exact test for biased strand distribution *p*-value = 2.68e-03 | | | | | | | | | | | |
| Kolmogorov-Smirnov test that lower quality scores support variant *p*-value = 5.16e-01 | | | | | | | | | | | |

Only 100 of 3075 total aligned reads displayed.  

`CTGTTCCGTTTGGTCAACTAAAGACCATGAACCAGAACTCATGGCAAGCACGTAATGAAGCCTTACAGTTCCGTAATGAGGCTGAGACTTTCAGAAACCAAGCGGAGGGCTTTAAGAACGAGTCCAGTACCAACGCTACGAACACAAAGCAGTGGCGCGATGAGACCAAGGGTTTCCGAGACGAAGCCAAGCGGTTCAAGAATACGGCTGGTCAATACGCTACATCTGCTGGGAACTCTGCTTCCGCTGCGCATCAATCTGAGGTAAACGCTGAGAACTCTGCCACAGCATCCGCTAACTCTGCTCATTTGGCAGAACAGCAAGCAGACCGTGCGGAACGTGAGGCAGACAAGCTGGAAAATTACAATGGATTGGCTGGTGCAATTGATAAGGTAGATGGAACCAATGTGTACTGGAAAGGAAATATTCACGCTAACGGGCGCCTTTACATGACCACAAACGGTTTTGACTGTGGCCAGTATCAACAGTTCTTTGGTGGTGTCACTAATCGTTACTCTGTCATGGAGTGGGGAGATGAGAACGGATGGCTGATGTATGTTCAACGTAGAGAGTGGACAACAGCGATAGGCGGTAACAT  >  NC_001604.1/35066‑35663`  

                                                                                                                                                                                                                                                                                                            `|`                                                                                                                                                                                                                                                                                                           
`cTGTTCCGTTTGGTCAACTAAAGACCATGGACCAGAACTCATGGCAAGCACGTAATGAAGCCTTACAGTTCCGTAATGAGGCTGAGACTTTCAGAAACCAAGCGGAGGGCTTTAAGAACGAGTCCAGTACCAACGCTACGAACACAAAGCAGTGGCGCGATGAGACCAAGGGTTTCCGAGACGAAGCCGAGCAGTTCAAGAATACGGCTGGTCAATACGCTACATCTGCTGGGAACTCTGCTTCCGCTGCGCATCAATCTGAGGTAAACGCTGAGAACTCTGCCACAGCATCCGCTAActc                                                                                                                                                                                                                                                                                                           <  1:153201/301‑1 (MQ=255)`  
  `gTTCCGTTTGGTCAACTAAAGACCATGGACCAGAACTCATGGCAAGCACGTAATGAAGCCTTACAGTTCCGTAATGAGGCTTAGACTTTCAGAAACCAAGCGGAGGGCTTTAAGAACGAGTCCAGTACCAACGCTACGAACACAAAGCAGTGGCGCGATTCGACCAATGGTTTCCGAGACGAAGCCAAGCGGTTCAAGAATACGGCTGGTCAATACGCTACATCTGCTGGGAACTCTGCTTCCGCTGCGCATCAATCTGAGGTAAACGCTGAGAACTCTGCCACAGCATCCGCTAACTATg                                                                                                                                                                                                                                                                                                         >  1:388750/1‑301 (MQ=255)`  
     `ccGTTTCGTCAACATAAGACCATTGAACAGAACTCATGGCCAGCACGTAATGCAGCCTTACAATGCCGTAATGAGGCTTAGACTTTCAGAAACCAAGCGGAGGGCGTTAAGAACGAGTCCCGTAACAACGCTACGAACACCAAGCAGTGGCGAGATGATACCAAGGGTTTCCGAGACGAAGCCAAGCGGTTCAAGAATACGGAGGGTCAATACGCTACATCTGCTGGGAAATCTGCTTCCGCTGCGCAGCAATCTGAGGTAAACGCTGAGAACTCTGCCACAGCATCCGCTAACTATGCTc                                                                                                                                                                                                                                                                                                      <  1:397863/301‑1 (MQ=255)`  
           `ggTCAACTAAAGACCATGGACCAGAACTCATGGCAAGCACGTAATGAAGCCTTACAGTTCCGTAATGAGGCTGAGACTTTCAGAAACCAAGCGGAGGGCTTTAAGAACGAGTCCAGTACCAACGCTACGAACACAAAGCAGTGGCGCGATGAGACAAAGGGTTTCCGAGACGAAGCCGAGCGGTTCAAGAATACGGCTGGTCAATACGCTACATCTGCTGGGAACTCTGCTTCAGCTGCGCATCAATCTGAGGTAAACGCTGAGAACTCTGCCACAGCATCCGCCAACTATGCTCAATTgg                                                                                                                                                                                                                                                                                                >  1:95633/1‑301 (MQ=255)`  
                      `gACCATGGACCAGAACTCATGGCAAGCACGTAATGAGGCCTTACAGTTCCGTAATGAGGCTGAGACTTTCAGAAACCATGCGGAGGGCTTTAAGAACGAGTCCAGTACCAACTCTACGAACACAAAGCAGTGGCGCGATGAGACCAAGGGTTTCCGAGACGAAGCCAAGCGGTTCAAGAATACGGCTGGTCAATACGCTACAACTGCTGGGAACTCTGCTTCCGCTGCGCATAACTCTGAGGTAAACGCTGATAACTCTGGCACAGAATCCGCTAACTATGCTCATTTTGCAGAACagcaa                                                                                                                                                                                                                                                                                     >  1:485872/1‑301 (MQ=255)`  
                             `gaCCAGAACTCATGGCAAGCACGTAATGAAGCCTTACAGTTCCGTAATGAGGCTGAGACTTTCAGAAACCAAGCGGAGGGCTTTAAGAACGAGTCCAGTACCAACGCTACGAACACAAAGCAGTGGCGCGATGAGACCAAGGGTTTCCGAGACGAAGCCGAGCGGTTCAAGAATACGGCTGGTCAATACGCTACATCTGCTGGGAACTCTGCTTCCGCTGCGCATCAATCTGAGGTAAACGCTGAGAACTCTGCCACAGCATCCGCTAACTCTGCTCATTTGGCATAACAGCAAGCAGAcc                                                                                                                                                                                                                                                                              >  1:481738/2‑301 (MQ=255)`  
                                  `gAACTCATGGCAAGCACGTAATGAAGCCTTACAGTTCCGTAATGAGGCTGAAACTTTCAGAAACCAAGCGGAGGGCTTTAAGAACGAGTCCAGTACCAACGCTACGAACACAAAGCAGTGGCGCGATGAGACCAAGGGTTTCCGAGACGAAGCCAAGCGGTTAAAGAATACGGCTGGTCAATACGCTACATATGCTGGGAACTCTGCTTCCGCTGCGCATCAATCTGAGGTCAACGCTGAGAACTATGCCACAGCATCCGATAACTATGCTCATTTGGCAGAACAGCAAGCAGACCGTGCg                                                                                                                                                                                                                                                                         >  1:157457/1‑301 (MQ=255)`  
                                      `tCATGGCAAGCACGTAATGAAGCCTTACAGTTCCGTAATGAGGCTGAGACTTTCAGAAACCAAGCGGAGGGCTTTAAGAACGAGTCCAGTACCAACGCTACGAACACAAAGCAGTGGCGCGATGAGACCAAGGGTTTCCGAGACGAAGCCAAGCGGTTCAAGGATACGGCTGGTCAATACGCTACATCTGCTGGGAACTCTGCTTCCGCTGCGCATCAATCTGAGGTAAACGCTGAGAACTCTGCCACAGCATCCGCTAACTATGCTCATTTGGCAGAACAGCAAGCAGACCGTGCGGAAc                                                                                                                                                                                                                                                                     <  1:274241/301‑1 (MQ=255)`  
                                            `gctcggagatgtggaaaagagacagtCCGTAATGAGGCTGAGACTTTCAGAAACCAAGCGGAGGGCTTTAAGAACGAGTCCAGTACCAACGCTACGAACACAAAGCAGTGGCGCGATGAGACCAAGGGTTTCCGAGACGAAGCCGAGCGGTTAAAGAATACGGCTGGTCAATACGCTACATATGCTGGGAACTCTGCTTCCGCTGCGAATCAATCTGAGGTAAACGCTGAGAACTCTGCCACAGCATCCGCTAACTATGCTCATTTGGCAGAACAGCAAGCAGACCGTGCGGAACGTGAgg                                                                                                                                                                                                                                                               <  1:457759/276‑1 (MQ=255)`  
                                            `cAAGCACGTAATGAAGCCTTACAGTTCCGTAATGAGGCTGAGACTTTCAGAAACCAAGCGGAGGGCTTTAAGAACGAGTCCAGTACCAACGCTACGAACACAAAGCAGTGGCGCGATGAGACCAAGGGTTTCCGAGACGAAGCCAAGCGGTTCAAGAATATGGCTGGTCAATACGCTACATCTGCTGGGAACTCTGCTTCCGCTGCGCATCAATCTGAGGTAAACGCTGAGAACTCTGCCACAGCATCCGCTAACTATGCTCATTTGGCAGAACAGCAAGCAGACCGTGCGGAACGTGAgg                                                                                                                                                                                                                                                               >  1:129222/1‑301 (MQ=255)`  
                                             `gacagcGTAATGAAGCCTTACAGTTCCGTAATGAGGCGGAGACTTTCAGAAACCAAGGGGAGGGCTTTAAGAACGAGTCCAGTACCAACGCTACGAACACAAAGCAGTGGCGCGATGAGACCAAGGGTTTCCGAGACGAAGCCAAGCGGTTCAAGAATACGGCTGGTCAATACGCTACATCTGCTGGGAACTCTGCTTCCGCTGCGCATCAATCTGAGGTAAACGCTGAGAACTATGCCACAGCATCCGCTAACTATGCTCATTTGGCAGAACAGCAAGCAGACCGTGCGGAACGTGAGGc                                                                                                                                                                                                                                                              <  1:468107/296‑1 (MQ=255)`  
                                                `cACGTAATGAAGCCTTACAGTTCCGTAATGAGGCTGAGACTTTCAGAAACCAAGCGGAGGGCTTTAAGAACGAGTCCAGTACCAACGCTACGAACACAAAGCAGTGGCGCGATGAGACCAAGGGTTTCCGAGACGAAGCCGAGCGGTTCAAGAATACGGCTGGTCAATACGCTACATCTGCTGGGAACTCTGCTTCCGCTGCGCATCAATCTGAGGTAAACGCTGAGAACTCTGCCACAGCATCCGCTAACTATGCTCATTTGGCAGAACAGCAAGCAGAACGTGCGGAACGTGAGGCAGa                                                                                                                                                                                                                                                           >  1:223206/1‑301 (MQ=255)`  
                                                 `aaagagacaggCCTTACAGTTCCGTAAGGAGGCTGAGACTTTCAGAAACCAAGCGGAGGGCTTTAAGAACGAGTCCAGTACAAACGCTACGAACACAAAGCAGTGGCGCGATGAGACCAAGGGTTTCCGAGACGAAGCCGAGCGGTTCAAGAATACGGCTGGTCAATACGCTACATCTGCTGGGAACTCTGCTTCCGCTGCGCATCAATCTGAGGTAAACGCTGAGAACTCTGCCACAGCATCCGCTAACTCTGCTCATTTGGCAGAACAGCAAGCAGACCGTGCGGAACGTGAGGCAGAc                                                                                                                                                                                                                                                          <  1:447070/291‑1 (MQ=255)`  
                                                 `aCGTAATGAAGCCTTACAGTTCCGTAATGAGGCTGAGACTTTCAGAAACCAAGCGGAGGGCTTTAAGAACGAGTCCAGTACCAACGCTACGAACACAAAGCAGTGGCGCGATGAGACCAAGGGTTTCCGAGACGAAGCCGAGCGGTTCAAGAATACGGCTGGTCAATACGCTACATCTGCTGGGCACTCTGCTTCAGCTGCGCATCAATCTGAGGTAAACGCTGAGAACTATGCCACAGCATCCGCCAACTATGCTCATTTGGCAGAACAGCAAGCAGACCGTGCGGCACGTGAGGCAGAc                                                                                                                                                                                                                                                          >  1:330409/1‑301 (MQ=255)`  
                                                 `aCGTAATGAAGCCTTACAGTTCCGTAATGAGGCTGAGACTTTCAGAAACCAAGCGGAGGGATTTAAGAACGAGTCCAGTACCAACGCTACGAACACAAAGCAGTGGCGCGATGAGACCAAGGGTTTCCGAGACGAAGCCAAGCGGTTCAAGAATACGGCTGGTCAATACGCTACATCTGCTGGGAACTCTGCTTCCGCTGCGCATCAATCTGAGGTAAACGCTGAGAACTCTGCCACAGCATCCGCTAACTATGCTCATTTGGCAGAACAGCAAGCAGACCGTGCGGAACGTGAGGCAGAc                                                                                                                                                                                                                                                          <  1:40152/301‑1 (MQ=255)`  
                                                     `aaTGAAGCCTTACAGTTCCGTAATGAGGCTGAGACTTTCAGAAACCAAGCGGAGGGCTTTAAGAACGAGTCCAGTACCAACGCTACGAACACAAAGCAGTGGCGCGATGAGACCAAGGGTTTCCGAGACGAAGCCGAGCGGTTCAAGAATACGGCTGGTCAATACGCTACATCTGCTGGGAACTCTGCTTCCGCTGCGCATCAATCTGAGGTCAAAGCTGAGAACTCTGCCACAGCATCCGCTAACTCTGCTCATTTGGCAGAACAGCACGCAGACCGTGCGGAACGTGAGGCAGACAAGc                                                                                                                                                                                                                                                      >  1:90985/1‑301 (MQ=255)`  
                                                            `ccTTACAGTTCCGTAATGAGGCTGAGACTTTCAGAAACCAAGCGGAGGGCTTTAAGAACGAGTCCAGTACCAACGCTACGAACACAAAGCAGTGGCGCGATGAGACCAAGGGTTTCCGAGACGAAGCCAAGCGGTTCGAGAATACGGCTGGTCAATACGCTACATCTGCTGGGAACTCTGCTTCCGCTGCGCATCAATCTGAGGTAAACGCTGAGAACTCTGCCACAGCATCCGCTAACTATGCTCATTTGGCAGAACAGCAAGCAGACCGTGCGGAACGTGAGGCAGACAAGCTGGaaac                                                                                                                                                                                                                                               >  1:232457/1‑300 (MQ=255)`  
                                                                     `tCCGTAATGAGGCTGAGACTTTCAGAAACCAAGCGGAGGGCTTTAAGAACGAGTCCAGTACCAACGCTACGAACACAAAGCAGTGGCGCGATGAGACCAAGGGTTTCCGAGACGAAGCCGAGCGGTTCAAGAATACGGCTGGTCAATACGCTACATCTGCTGGGAACTCTGCTTCCGCTGCGCATCAATCTGAGGTACACGCTGAGAACTCTGCAACAGCATCCGCTAACTATGCTCATTTGGCAGAACAGCAAGCAGACCGTGCGGAACGTGAGGCAGACAAGCTGGAAAATTACAATgg                                                                                                                                                                                                                                      >  1:438963/1‑301 (MQ=255)`  
                                                                     `tCCGTAATGAGGCTGAGACTTTCAGAAACCAAGCGGAGGGCTTTAAGAACGAGTCCAGTACCAACGCTACGAACACAAAGCAGTGGCGCGATGAGACCAAGGGTTTCCGAGACGAAGCCAAGCGGTTCAAGAATATGGCTGGTCAATACGCTACATCTGCTGGGAACTCTGCTTCCGCTGCGCATCAATCTGAGGTAAACGCTGAGAACTCTGCCACAGCATCCGCTAACTATGCTCATTTGGCAGAACAGCAAGCAGACCGTGCGGAACGTGAGGCAGACAAGCTGGAAAATTACAATgg                                                                                                                                                                                                                                      >  1:82956/1‑301 (MQ=255)`  
                                                                        `gTAATGAGGCTGAGACTTTCAGAAACCAAGCGGAGGGCTTTAAGAACGAGTCCAGTACCAACGCTACGAACACAAAGCAGTGGCTCGATGAGACCAAGGGTTTCCGAGACGAAGCCAAGCGGTTCAAGAATATGGCTGGTCAATACGCTACATCTGCTGGGAACTCTGCTTCCGCTGCGCATCAATCTGAGGTAAACGCTGAGAACTCTGCCACAGCATCCGCTAACTATGCTCATTTGGCAGAACAGCAAGCAGACCGTGCGGAACGTGCGGCAGACAAGCTGGAAAATTACAATGGAtt                                                                                                                                                                                                                                   >  1:191832/1‑301 (MQ=255)`  
                                                                        `gTAATGAGGCTGAGACTTTCAGAAACCAAGCGGAGGGCTTTAAGAACGAGTCCAGTACCAACGCTACGAACACAAAGCAGTGGCGCGATGAGACCAAGGGTTTCCGAGACGAAGACAAGCGGTTCAAGAATACGGCTGGTCAATACGCTACATCTGCTGGGAACTCTGCTTCCGCTGCGCATCAATCTGAGGTAAACGCTGAGAACTCTGCCACAGCATCCGCTAACTATGCTCATTTGGCAGAACAGCAAGCAGACCGTGCGGAACGTGAGGCATACAAGCTGGAAAATTACAATGGAtt                                                                                                                                                                                                                                   >  1:431441/1‑301 (MQ=255)`  
                                                                            `tGAGGCTGAGACTTTCAGAAACCAGGCGGCGGGCTTTAAGAACGAGTCCAGTACCAACGCTACTAACACAAATCAGTGTCGCGATGAGACCAAGGGTTTCCGAGACGAAGCCGAGCGGTTCAAGACTACGGCTGGTCAATACGCTACATCTGCTGGGAACTCTGCTTCCGCTGCGCAGCAATCTGAGGTAAACGCTGAGAACTCTGCCACAGCATCCGCTAACTATGCTCATTTGGCATAACAGCAAGCAGACCGTGCGGAACGTGAGGCAGACAAGCTGGAAAATTACAATGGATTGGCt                                                                                                                                                                                                                               <  1:498256/301‑1 (MQ=255)`  
                                                                                            `aGAAAACAAGCGGAGGGCTTTAAGAACGAGGCCAGTACCAACGCTACGAACACAAAGCAGTGGCGCGATGAGACCAAGGGTTTCCGCGACGAAGCCAAGCGGTTCAAGAATACGGCTGGTCAATACGCTACATCTGCTGGGAACTCTGCTTCCGCTGCGCATCAATCTGAGGTAAACGCTGAGAACTCTGCCACAGCATCCGCTAACTATGCTCATTTGGCAGAACAGCAAGCAGACCGTGCGGAACGTGAGGCAGACAAGCTGGAAAATTACAATGGATTGGCTGGTGCAATTGATAAgg                                                                                                                                                                                                               <  1:70765/301‑1 (MQ=255)`  
                                                                                                 `ccAAGCGTAGGGCTTTAAGAACGAGTCCAGTACCAACGCTACGAACACAAAGCAGTGGCGCGATGAGACCAAGGGTTTCCGAGACGAAGCCGAGCGGTTCAAGAATATGGCTGGTCAATACGCTACATCTGCTGGGAACTCTGCTTCCGCTGCGCATCAATCTGAGGTAAACGCTGAGAACTCTGCCACAGCATCCGCTAACTCTGCTCATTTGGCAGAACAGCAAGCAGACCTTTCGGAACGTGAGGCAGACAAGCTGGAAAATTACAATGGATTGGCTGGTGCAATTGATAAGGTAGAt                                                                                                                                                                                                          >  1:378280/1‑301 (MQ=255)`  
                                                                                                      `gggAAGGCTTTAAGAACGAGTCCAGTACCAAAGCTACGAACACAAGGCAGCGGTGCGATGAGACCAAGGGTTTCCGAGACGAAGCCAAGCGGTTCAAGAATACGGCTGGTCAATACGCTACATCTGCTGGGAACTCAGCGTCCGCTGCGCATCAATCTGAGGTAAACGCTGAGAACTATGCCACAGCATCCGCTAACTATGCTCATTTGGCAGAACAGCAAGCAGACCTTGCGGAACGTGAGTCAGACAAGCTGGAAAATTACAATGGATTGGCTGGTGCAATTGATAAGGTAGATGGAAc                                                                                                                                                                                                     <  1:147591/300‑1 (MQ=255)`  
                                                                                                       `gacaggCTTTAAGAACGAGTCCAGTACCAACGCTACGAACACAAAGCAGTGGCGCGATGAGACCAAGGGTTTCCGAGACGAAGCCAAGCGGTTCAAGAATACGGCTGGTAAATACGCTACATCTGCTGGGAACTCTGCTTACGCTGCGCATCAATATGAGGTAAACGCTGAGAACTATGCCACAGCATCCGCTAACTATGCTCATTTGGCAGAACAGCAAGCAGACCGTGCGGAACGTGAGGCAGACAAGCTGGAAAATTACAATGGATTGGCTGGTGCAATTGATAAGGTAGATGGAAcc                                                                                                                                                                                                    <  1:183482/297‑1 (MQ=255)`  
                                                                                                       `agagtcagttAAGAACGAGTCCATTACCAACGCTACGAACACAAAGCAGTGGCGCGATGAGACCAAGGGTTTCCGAGACGAAGCCGAGCGGTTCAAGAATACGGCTGGTCAATACGCTACATCTGCTGGGAACTCTGCTTCCGCTGCGCATCAATCTGAGGTAAACGCTGAGAACTCTGCCACAGCATCCGCTAACTCTGCTCATTTGGCAGAACAGCAAGCAGACCGTGCGGAACGTGAGGCAGACAAGCTGGAAAATTACAATGGATTGGCTGGTGCAATTGATAAGGTAGATGGAAcc                                                                                                                                                                                                    <  1:209941/293‑1 (MQ=255)`  
                                                                                                          `gggTTTTAAGAACGAGTCCAGTACCAACGCTACGAACACAAAGCAGTGGCGCGATGAGACCAAGGGTTTCCGAGACGAAGCCAAGCGGTTCAAGAATACGGCTGGTCAATACGCTACATCTGCTGGGAACTCTGCTTCCGCTGCACATCAATCTGAGGTAAACGCTGAGAACTCTGCCACAGCATCCGCTAACTATGCTCATTTGGCAGAACAGCAAGCAGACCGTGCGGACCGCGAGGCAGACAAGCTGGACAATTACAATGGATTGGCTGGTGCAATTGATAAGGTAGATGGAACCAAt                                                                                                                                                                                                 >  1:38399/1‑301 (MQ=255)`  
                                                                                                           `ggCTTTAAGAACGAGTCCAGTACCAACGCTACGAACACAAAGCAGTGGCGCGATGAGCCCAAGGGTTTCCGAGACGAAGCCGAGCGGTTCAAGAATACGGCTGGTCAATACGCTACATCTGCTGGGAACTCTGCTTCCGCTGCGCATCAATCTGAGGTAAACGCTGAGAACTATGCCACAGCATCCGCTAACTCTGCTCATTTGGCCGCACAGCAAGCAAACCGTTCTGAACGGGAGGCCGACAAGCTGGAAACTTACAATGGATTGGCTGGTTCAATTGATAAGGTAAATGGAACCAAtt                                                                                                                                                                                                >  1:256643/1‑300 (MQ=255)`  
                                                                                                           `ggCTTTAAGAACGAGTCCAGTACCAACGCTACGAACACAAAGCAGTGGCGCGATGAGACCAAGGGTTTCCGAGACGAAGCCGAGCGGTTCAAGAATACGGCTGGTCAATACGCTACATCTGCTGGTAACTCTGCTTCCGCTGCGCATCAATCTGAGGTAAACGCTGAGAACTCTGCCACAGCATCCGCTAACTATGCTCATTTGGCAGAACAGCAAGCAGACCGTGCGGAACGTGAGGCAGACAAGCTGGAAAATTACAATGGATTGGCTGGTGCAATTGATAAGGTAGATGGAACCAAtt                                                                                                                                                                                                >  1:435543/1‑300 (MQ=255)`  
                                                                                                           `ggCTTTAAGAACGAGTCCAGTACCAACGCTACGAACACAAAGCAGTGGCGCGATGAGACCAAGGGTTTCCGAGACGAAGCCGAGCGGTTCAAGAATACGGCTGGTCAATACGCTACATCTGCTGGGAACTCTGCTTCCGCTGCGCATCAATCTGAGGTAAACGCTGAGAACTCTGCCACAGCATCCGCTAACTATGCTCATTTGGCAGAACAGCAAGCAGACCGTGCGGAACGTGAGGCAGACAAGCTGGAAAATTACAATGGATTGGCTGGTGCAATTGATAAGGTAGATGGAACCAAtg                                                                                                                                                                                                >  1:345553/1‑301 (MQ=255)`  
                                                                                                           `ggCTTTAAGAACGAGTCCAGTACCAACGCTACGAACACAAAGCAGTGGCGCGATGAGACCAAGGGTTTCCGAGACGAAGCCGAGCGGTTCAAGAATACGGCTGGTCAATACGCTACATCTGCTGGGAACTCTGCTTCCGCTGCGCATCAATCTGAGGTAAACGCTGAGAACTCTGCCACAGCATCCACTAACTCTGCTCATTTGGCAGAACAGCAAGCAGACCGTGCGGAACGTGAGGCAGACAAGCTGGAAAATTACAATGGATTGGCTGGTGCAATTGATAAGGTAGATGGAACCGAtg                                                                                                                                                                                                >  1:357910/1‑301 (MQ=255)`  
                                                                                                            `gCTTTAAGAACGAGTCCAGTACCAACGCTACGAACACAAAGCAGTGGCGCGATGAGACCAAGGGCTTCCGAGACGAAGACAAGCGGTTCAAGAATATGGCTGGTCAATACGCTACATCTGCTGGGAACTCTGCTTCCGCTGCGCATCAATCTGAGGTAAACGCTGAGAACTCTGCCACAGCATCCGCTAACTATGCTCATTTGGCAGAAAAGCAAGCAGACCGGGCGGAACGTGAGGCAGACGAGCGGGAAAAGTACAATGGCTTGGATGGTGGGAATACTAAGGTAGATGGAAACaaagc                                                                                                                                                                                               >  1:245811/1‑298 (MQ=255)`  
                                                                                                             `cTTTAAGAACGAGTCCAGTACCAACGCTACGAACACAAAGCAGTGGCGCGATGAGACCAAGGGTTTCCGAGACGAAGCCGAGCGGTTCAAGAATACGGCTGGTCAATACGCTACATCTGCTGGGAACTCTGCTTCCGCTGCGCATCAATCTGAGGTAAACGCTGAGAACTCTGCCACAGCATCCGCTAACTCTGCTCATTTGGCAGAACAGCAAGCAGACCGTGCGGAACGTGAGGCAGACAAGCTGGAAAATTACAATGGATTGGCTGGTGCAATTGATAAGGTAGATGGAAccctgtct                                                                                                                                                                                              >  1:393857/1‑295 (MQ=255)`  
                                                                                                                   `gtgggctcggagatgtggataagagacagcaAAGAAGTGGCGCGATGAGACCAAGGGTTTCCGAGACGAAGCCGAGCGGTTCAAGAATACGGCTGGTCAATACGCTACATCTGCTGGGAACTCTGCTTCCGCTGCGCATCAATCTGAGGTAAACGCTGAGAACTCTGCCACAGCATCCGCTAACTATGCTCATTTGGCAGAACAGCAAGCAGACCGTGCGGAACGTGAGGCAGACAAGCTGGAAAATTACAATGGATTGGCTGGTGCAATTGATAAGGTAGATGGAACCAATGTGTACTgg                                                                                                                                                                                        <  1:170126/272‑1 (MQ=255)`  
                                                                                                                   `agacaggTCCAGTACCAACGCTACGAACACAAAGCAGTGGCGCGATGAGACCAAGGGGTTCCGAGACGAAGCCGAGCGGTTCAAGAATACGGCTGGTCAATACGCTACATCTGCTGGGAACTCTGCTTCCGCTGCGCATCAATCTGAGGTAAACGCTGAGAACTCTGCCACAGCATCCGCTAACTATGCTCATTTGGCAGAACAGCAAGCAGACCGTGCGAAACGTGAGGCAGACAAGCTGGAAAATTACAATGGATTGGCTGGTGCAATTGATAAGGTAGATGGAACCAATGTGTACTgg                                                                                                                                                                                        <  1:413917/295‑1 (MQ=255)`  
                                                                                                                   `aaaCGAGTCCAGTACCAACGCTACGAACACAAAGCAGGTGCGCGATGAGACCAAGGGTTTCCGAGACGAAGCCAAGCGGTTCAAGGATACGGCTGGTCAATACGCTACATCTTCTGTGAACTATGCTTCCGCTGCGCATCAATCTGCGGTAAACGCTGAGAACTCGGCCACAGCATCCGCTAACTCTGCTCATTTGGCAGAACAGCAAGCAGACCGTGCGGAACGTGCGGCAGACAAGCTGGAAAATTACAATGGATTGGCTGGTGCAATTGATAAGGTAGATGGAACCAATGTGTACTgg                                                                                                                                                                                        <  1:61890/300‑1 (MQ=255)`  
                                                                                                                     `aCGAGTCCAGGACCAACGATACGAACACAAAGCAGTGGCGGGATGAGACACAGGGTTTCAGAGACGGAGCCAAGCGGTTCAAGAATACGGCTGGTCAATACGCTACATCTTCTGGGAACTCTGCTTCCGCTGCGAATCAATCTGAGGTAAACGCTGAGAACTCTGCCACAGCAGCCGCTAACTATGCTCATTTGGCAGAACAGCAAGCAGACCGTGCGGAACGTGAGGCAGACAAGCTGGAAAATTACAATGGATTGGCTGGTGCAATTGATAAGGTAGATGGAACCAATGTGTACTggaa                                                                                                                                                                                      <  1:304783/301‑1 (MQ=255)`  
                                                                                                                          `tCCAGTACCAACGCTACGAACACAAAGCAGTGGCGCGATGAGACCAAGGGTTTCCGAGATGAAGCCGAGCGGTTCAAGAATACGGCTGGTCAATACGCTACATCTGCTGGGAACTCTGCTTCCGCTGCGCATCAATCTGAGGTAAACGCTGAGAACTCTGCCACAGCATCCGCTAACTATACTCATTTGGCAGAACAGCAAGCAGACCGTGCGGAACGTGAGGCAGACAAGCTGGAAAATTACAATGGATTGGCTGGTGCAATTGATAAGGTAGATGGAAACAATGTGTAcctgtctctta                                                                                                                                                                                 >  1:124819/1‑291 (MQ=255)`  
                                                                                                                               `taccacctacacgaaacgaaaGCAGTGTCGCGAGCGGACCAAGGGTTTCCGAGACGAAACAGAGCGGTTCAAGACCACGGCTAGACAATACGCTATAGCTGTTGGGGACGCTGCTTCCGCTGCGCATCAATCGTAGGTAAACGCTGAGAACCATGCCAATGCATCCGCGAACTCGGCTCATTAGGCAGAACAGCAAGCAGACCGTGCGGAACGTGAGGAAGACAAGCTGGAAAATTACAATGGATTGGCTGGTGCAATTGAGAATGTAGATGGAACCGATGTGTACTGGAAAGGAAATAtt                                                                                                                                                                            <  1:33449/283‑1 (MQ=255)`  
                                                                                                                               `tACCAACGCTACGAACACAAAGCAGTGGCGCGATGAGACCAAGGGGTTCCGAGACGAAGCCAAGCGGTTCAAGAATACGGCTGGTCAATACGCTACATCTGCTGGGAACTCTGCTGCCGCTGCGCATCAGTCTGAGGTAAACGCTGAGAACTCTGCCACAGCATCAGCTAACTATGCTAATTGGGCAGACAAGCAAGCAGACCGTGCGGAACGTGAGGCAGAGAAGATGGGAAATTACAAGGGATTGGCTGGTGCAATTGATAAGGAAGATGGAACAACGGTGGACTGGAAAGGAAaaatg                                                                                                                                                                            >  1:111779/1‑300 (MQ=255)`  
                                                                                                                                `aCCAACGCTACGAACACAAAGCAGTGGCGCGATGAGACCAAGGGTTTCCGAGACGAAGCCGAGCGGTTCAAGAATACGGCTGGTCAATACGCTACATCTGTTGGGAACTCTGCTTCCGCTGCGCATCAATCTGAGGTAAACGCTGAGAACTCTGCCACAGCATCCGCTAACTCTGCTCATTTGGCAGAACAGCAAGCAGACCGTGCGGAACGTGAGGCAGACAAGCTGGAAAATTACAATGGATTGGCTGGTGCAATTGATAAGGTAGATGGAACCAATGTGTACTGGAAAGGAAATATTc                                                                                                                                                                           >  1:150341/1‑301 (MQ=255)`  
                                                                                                                                       `cTACGAACACAAAGCAGTGGCGCGATGAGACCAAGGGTTTCCGAGACGAAGCCAAGCGGTTCAAGAATACGGCTGGTCAATACGCCACATCTGCTGGGAACTCTGCTTCCGCTGCGCATCAATCTGAGGTAAACGCTGAGAACTATGCCACAGCATCCGCTAACTATGCTCCTTTGGCAGAACAGCAAGCAGACCGTGCGGAACGTGAGGCAGACAAGCTGGAAAATTACAATGGATTGGCTGGTGCCATTGATAAGGTAGATGGAACAAATGTGTACTGGCAAGGTGATATTcctgtctc                                                                                                                                                                    >  1:79528/1‑294 (MQ=255)`  
                                                                                                                                         `aCGAACACAAAGCAGTGGCGCGATGAGACCAAGGGTTTCCGAGACGAAGCCAAGCGGTTCAATAATCCGGCTGGTCAATACGCTACATCTGCTGGGAACTCTGCTTCCGCTGCGCATCAATCTGAGGTAAACGCTGAGACCTCTGCCACAGCATCCGCTAACTATGCTCATTTGGCGAAACAGCCAGCAGACCGTGCGGAACGTGAGGCAGACAAGCTGGAAAATTACAATTGAATGGCTGGTGCAATTGATAAGGTAGATGGAACCAATGTGTACTGGAAAGGAAATATTCACGATAACg                                                                                                                                                                  >  1:503724/1‑301 (MQ=255)`  
                                                                                                                                                      `aGTGGCGCAATGAGACCAAGGGTTTCCGAGACGAAGCCAAGCGGTTCAAGAATACGGCTGTTCAATACGCGAAGTCTGCTGGGAACTCTGCTTCCGCTGCGCATCAAACTGAAGTAAACGCTGAGAACTCTGCCACAGCATCCGCTAACTATGCTTATTTGGCAGAACAGCAAGCAGACCGTGCGGAACGTGAGGCAGACAAGCTGGAAAATTACAATGGATTGGCTGGTGCAATTGATAAGGTAGATGGAACCAATGTGTACTGGAAAGGAAATATTCACGCTAACGGGCGCCTTTACAt                                                                                                                                                     <  1:373405/301‑1 (MQ=255)`  
                                                                                                                                                       `gTGGCGCGATGAGACCAAGGGTTTCCGAGACGAAGCCAAGCGGTTCAAGAATACGGCTGGTCAATACGCTACATCTGCTGGGAACTCTGCTTCCGCTGCGCATCAATCTGAGGTAAACGCTGAGAACTCTGCAACAGCATCCGCTAACTATGCTCTTTTGGCAGAACAGCAAGCAGACCGTGCGGAACGTGAGGCAGACAAGCTGGAAAATTACAATGGATTGGCTGGTGCAATTGATAAGGTAGATGGAACCAATGTGTACTGGAAAGGAAATATTCACGCTAACGGGCGCCTTTACCTg                                                                                                                                                    >  1:20466/1‑301 (MQ=255)`  
                                                                                                                                                             `cgATGAGACCAAGGGTTTCCGAGACGAAGCCGAGCGGTTCAAGAATACGGCTGGTAAATACGCTACATCTGCTGGGAACTCTGCTTCCGCTGCGCATCAATCTGAGGTAAACGCTGAGAACTCTGCCACAGCATCCGCTAACTATGCTCATTTGGCAGACCAGCAAGCAGACCGTGCGGAACGTGAGGCAGACAAGCTGGAAAATTACAATGGATTGGCTGGTGCAATTTATAAGGTAGATGGAACCAATGTGTACTGGAAAGGAAATATTAACGCTAACGGGCGCCTTTACATgctgtct                                                                                                                                              >  1:315030/1‑295 (MQ=255)`  
                                                                                                                                                                   `gaCCAAGTGTTTCCGAGACGAAGCCGAGCGGTTCAAGAATACGGCTGGTCAATACGCTACATCTGCTGGGAACTCTGCTTCCGCTGCGCATCAATCTGAGGTATACGCTGAGAACTCTGCCACAGCATCCGCTAACTATGCTCATTTGGCAGAACAGCAAGCAGACCGTGCGGAACGTGAGGCAGACAAGCCGGAAAATTACAATGGATTGGCTGGTGCAATTGATAAGGTAGATGGAACCAATGTGTACTGGAAAGGAAATATTCACGCTAACGGGCGCATTTACATGACCACAAACGGt                                                                                                                                        >  1:291393/1‑301 (MQ=255)`  
                                                                                                                                                                   `gaCCAAGGGTTTCCGAGACGAAGCCGAGCGGTTCAAGAATACGGCTGGTCAATACGCTACATCTGCTGGGAACTCTGCTTCTGCTGCGCATCAATCTGAGGTAAACGCTGAGAACTCTGCCACAGCATCCGCTAACTATGCTCATTTGGCAGAACAGCAAGCAGACCGTGCGGAACGTGACGCAGACAAGCTGGAAAATTACAATGGATTGGCTGGTGCAATTGCTAAGGTCGATGGAACCAATGTGTAGTGGAAAGGATATATTCACGCTAACGGGCGCCTTTACTTGACCACACACgga                                                                                                                                        >  1:368567/1‑300 (MQ=255)`  
                                                                                                                                                                   `gaCCAAGGGTTTCCGAGACGAAGCCGAGCGGTTCAAGAATACGGCTGGTCAATACGCTACATCTGCTGGGAACTCTGCTTCCGCTGCGCATCAATCTGAGGTAAACGCTGAGAACTCTGCCACAGCATCCGCTAACTCTGCTCATTTGGCAGAACAGCAAGCAGACCGTGCGGAACGTGAGGCAGACAAGCTGGAAAATTACAATGGATTGGCTGGTGCAATTGATAAGGTAGATGGAACCGATGTGTACTGGAAAGGAAATATTCACGCAAACGGGCGCCTTTACATGACCACAAACggg                                                                                                                                        >  1:34046/1‑300 (MQ=255)`  
                                                                                                                                                                          `tcggagatgtgtataagagacaggTTCAAGAATACGGCTGGTCAATACGCTACATCTGCTGGGAACTCTGCTTCCGCTTCGCATCAATCTGAGGTAAACGCTGAGAACTCTGCCACAGCATCCGCTAACTATGCTCATTTGGCAGAACAGCAAGCAGACCGTGCGGAACGTGAGGCAGACAAGCTGGAAAATTACAATGGATTGGCTGGTGCAATTGATAAGGTAGATGGAACCAATGTGTACTGGAAAGGAAATATTCACGCTAACGGGCGCCTTTACATGACCACAAACGGTTTTGACt                                                                                                                                 <  1:287168/279‑1 (MQ=255)`  
                                                                                                                                                                           `gTTTCCGAGACGAAGCCAAGCGGTTCAAGAATAGGGCTGGTCAATACGCTACATCTGCTGGGAACTCTGCTTCCGCTGGGCATCAATCTGAGGTAAACGCTGAGAACCCTGCCACAGCATCCGCTAACTATGCTCATTTGGCAGAACAGCAAGCAGACCGTGCGGAACGTGAGGCAGACAAGCTGGAAAATTACAATGGATTGGCTGGTGCAATTGATAAGGTAGATGGAACCAATGTGTACTGGAAAGGAAATATTCACGCTAACGGGCGCCTTTACATGACCACAAACGGTTTTGACtg                                                                                                                                <  1:266197/301‑1 (MQ=255)`  
                                                                                                                                                                             `ttCCGAGACGAAGCCGAGCGGTTCAAGAATACGGCTGGTCAATACGCTACATCTGCTGGGAACTCTGCTTCCGCTGCGCATCAATCTGAGGTAAACGCTGAGAACTCTGCCACAGCATCCGCTAACTATGCTCATTTGGCAGAACAGCAAGCAGACCGTGCGGAACGTGAGGCAGACAAGCTGGAAAATTACAATGGATTGGCTGGTGCAATTGATAAGGTAGATGGAACCAATGTGTACTGGAAAGGAAATATTCACGCTAACGGGCGCCTTTACATGACCACAAACGGTTTTGACtgtg                                                                                                                              >  1:300701/1‑301 (MQ=255)`  
                                                                                                                                                                                   `gaCGAAGCCGAGCGGTTCAAGAATACGGCTGGTCAATACGCTACATCTGCTGGGAACTCTGCTTCCGCTGCGCATCAATCTGAGGTAAACGCTGAGAACTCTGCCACAGCATCCGCTAACTCTGCTCATTTGGCAGAACAGCAAGCAGACCGTGCGGAACGTGAGGCAGACAAGCTGGAAAATTACAATGGATTGGCTGGTGCAATTGATAAGGTAGATGGAACCGATGTGTACTGGAAAGGAAATATTCACGCTAACTGGCGACTTTACATGACCACAAACGGTTTTGACTGTGGCCAGt                                                                                                                        >  1:349299/1‑301 (MQ=255)`  
                                                                                                                                                                                   `gaCGAAGCCAAGCGGTTCAAGAATATGGCTGGTCAATACGCTACATCTGCTGGGAACTCTGCTTCCGCTGCGCATCAATCTGAGGTAAACGCTGAGAACTCTGCCACAGCATCCGCTAACTATGCTCATTTGGCAGAACAGCAAGCAGACCGTGCGGAACGTGAGGCAGACAAGCTGGAAAATTACACTGGATAGGCTGGTGCAATTGATAAGGTAGATGGAACCAATGTGTACTGGAAAGGAAATATTCACGCTAACGGGCGCCTTTACATGACCACAAACGGTTTTGACTGTGGCCAGt                                                                                                                        >  1:318399/1‑301 (MQ=255)`  
                                                                                                                                                                                     `tataagagacaggTTCAAGAATACGGCTGGTCAATACGCTACATCTGCTGGGAACTCTGCTTCCGCTGCGCATCAATCTGAGGTAAACGCTGAGAACTCTGCCACAGCATCCGCTAACTATGCTCATTTGGCAGAACAGCAAGCAGACCGTGCGGAACGTGAGGCAGACAAGCTGGAAAATTACAATGGATTGGCTGGTGCAATTGATAAGGTAGATGGAACCAATGTGTACTGGAAAGGAAATATTCACGCTAACGGGCGCCTTTACCTGACCACAAACGGTTTTGACTGTGGCCAGTAt                                                                                                                      <  1:236537/290‑1 (MQ=255)`  
                                                                                                                                                                                       `aaGCCAAGCGGTTCAAGAATACGGCTGGTCAATACGCTACATCTGCTGGGAACTCTGCTTCCGCTGCGCATCAATCTGAGGTAAACGCTGAGAACTCTGCCACAGCATCCGCTAACTATGCTCATTTGGCAGAACAGCAAGCAGACCGTGCGGAACGTGAGGCAGACAAGCTGGAAAATTACAATGGATTGGCTGGTGCAATTGATAAGGTAGATGGAACCAATGTGTACTGGAAAGGAAATATTCACGCTAACGGGCGCCTTTACATGACCACAAACGGTTTTGACTGTGGCCAGTATCa                                                                                                                    >  1:86163/1‑301 (MQ=255)`  
                                                                                                                                                                                              `gCGGGTCAATAATCCGGCTGGTCAATACGATACATCTGCTGGGAACTCTGCTTCCGCTGCGAATCAATCTGAGGTAAACGCTGAGAACTCTGCCACAGCATCCGCTAACTATGCTAATTTGGCAGAACAGCAAGCAGACCGTGCGGAACGTGAGGCAGACAAGCTGGAAAATTACAATGGATTGGCTGGTGCAATTGATAAGGTAGATGGAACCAATGTGTACTGGAAAGGAAATATTCACGCTAACGGGCGCCTTTACATGACCACAAACGGTTTTGACTGTGGCCAGTATCAACAGTTc                                                                                                             <  1:323591/301‑1 (MQ=255)`  
                                                                                                                                                                                                 `gTTCAAGAATACGGCTGGTCAATACGCTACATCTGCTGGGAACTCTGCTTCCGCTGCGCATCAATCTGAGGTAAACGCTGAGAACTCTGCCACAGCATCCGCTAACTATGCTCATTTGGCAGAACAGCAAGCAGACCGTGCGGAACGTGAGGCAGACAAGCTGGAAAATTACAATGGATTGGCTGGTGCAATTGATAAGGTAGATGGAACCAATGTGTACTGGAAAAGAAATATTCACGCTAACGGGCGCCTTTACCTGACCACAAACGGTTTTGACTGTGGCCAGTATCAACAGTTCttt                                                                                                          >  1:477371/1‑301 (MQ=255)`  
                                                                                                                                                                                                 `gTTCAAGAATACGGCTGGTCAATACGCTACATCTGCTGGGAACTCTGCTTCCGATGCGCATCAATCTGAGGTAAACGCTGAGAACTCTGCCACAGCATCCGCTAACTATGCTCATTTGGCAGAACAGCACGCAGACCGTGCGGAACGTGAGGCAGACAAGCTGGAAAATTACAATGGATTGGCTGGTGCAATTGATAAGGTAGATGGAACAAATGTGTACTGGAAAGGAAATATTCACGCTAACGGGCGCCTTTACATGACCACAAACGGTTTTGACTGTGGCCAGTATCAACAGTTCttt                                                                                                          >  1:491905/1‑301 (MQ=255)`  
                                                                                                                                                                                                    `cAAGAATACGGCTGGTCAATACGCTACATCTGCTGGGAACTCTGCATGCGCTGCGCATCAATCTGAGGTAAACGCTGAGAACTGTGACACAGCATCCGCTAACTATGCTCATTTGGCAGAACAGCAAGCAGACCGTGCGGAACGTGAGGCAGAACAGCTGGAAAAATACAATGGATTTGCTGGTTCACTTGATAAGGAAGATGGAACCAATGGGTACTGGAAAGGAAATATTCACGCTAACGGGCGCCTTTAACTGACCACAAAAGGGTTTGAATGAGGCAAGtgacaaacggacattggt                                                                                                       >  1:510273/1‑284 (MQ=255)`  
                                                                                                                                                                                                       `gAATATGGCTGGTCAATACGCTACATCTGCTGGGAACTCTGCTTCCGCTGCGCATCAATCTGAGGTAAACGCTGAGAACTCTGCCACAGCATCCGCTAACTATGCTCATTTGGCATAACAGCAAGCAGACCGTGCGGAACGTGAGGCAGACAAGCTGGAAAATTACAATGGATTGTCTGGTGCAATTGATAAGGTAGATGGAACCAATGTGTACTGGAAAGGAAATATTCACGCTAACGGGCGCCTTTACATGACCACAAACGGTTTTGACTGTGGCCAGTATCAACAGTTCTTTggtggt                                                                                                    >  1:187766/1‑301 (MQ=255)`  
                                                                                                                                                                                                       `gAATACGGCTGGTCAATACGCTACATCTGCTGGGAACTCTGCTTCCGCTGCGCATCAATCTGAGGTAAACGCTGAGAACTCTGCCACAGCATCCGCTAACTATGCTCATTTGGCAGAACAGCAAGCAGACCGTGCGGAACGTGAGGCAGACAAGCTGGAAAATTACAATGGATTGGCTGGTGCAATTGATAAGGTAGATGGAACCAATGTGTACTGGAAAGGAAATATTCACGCTAACGGGCGCCTTTACCTGACCACAAACGGTTTTGACTGTGGCCAGTATCAACAGTTCTTTggtggt                                                                                                    >  1:1441/1‑301 (MQ=255)`  
                                                                                                                                                                                                       `gAATACGGCTGGTCAATACGCTACATCTGCTGGGAACTCTGCTTCCGCTGCGCATCAATCTGAGGTAAACGCTGAGAACTCTGCCACAGCATCCGCTAACTATGCTCATTTGGCAGAACAGCAAGCAGACCGTGCGGAACGTGAGGCAGACAAGCTGGAAAATTACAATGGATTGGCTGGTGCAATTGATAAGGTAGATGGAACCAATGTGTACTGGAAAGGAAATATTAACGCTAACGGGCGCCTTTACATGACCAAAAACGGTTTTGACTGTGGCCAGTATCAACAGTTCCTtggctgg                                                                                                    >  1:296408/1‑297 (MQ=255)`  
                                                                                                                                                                                                       `gAATACGGCTGGTCAATACACTACATCTGCTGGGAACTCTGCTTCCGCTGCGAATCAATCTGAGGTAAACGCTGAGAACTCTGCCACAGCATCCGCTAACTATGCGCATTTGGCAGAACAGCAAGCAGACCGTGCGGAACGTGAGGCAGACAAGCTGGAAAATTACAATGGATTGGCTGGTACAATTGATAAGATAGATGGAACCAATGTGTACTGGAAAGGAAATATTCACGCTAACGGGCGCCTTTACCTGACCACAAACGGTTTTGACTGTGGCCAGTACAAACAGTTCTTtggctgt                                                                                                    >  1:261570/1‑297 (MQ=255)`  
                                                                                                                                                                                                                 `ggTCAATACGCTACATCTGCTGGGAACTCTGCTTCCGCTGCGCATCAATCTGAGGTAAACGCTGAGAACTCTGCCACAGCATCCGCTAACTATGCTCATTTGGCAGAACAGCAAGCAGACCGTGCGGAACGTGAGGCAGACAAGCTGGAAAATTACAATGGATTGGCTGGTGCAATTGATAAGGTAGATGGAACCAATGTGTACTGGCAAGGAATTATTCACGCTAACGGGCGCCTTTACATGACCACAAACGGTTTTGACTGTGGGCGGTATCAACCGTCCTTTGGTTGTGTCAccctgt                                                                                          >  1:103442/1‑296 (MQ=255)`  
                                                                                                                                                                                                                 `ggTCAATACGCTACATCTGCTGGGAACTCTGCTTCCGCTGCGCATCAATCTGAGGTAAACGCTGAGAACTCTGCCACAGCATCCGCTAACTATGCTCATTTGGCAGAACAGCAAGCAGACCGTGCGGAACGTGAGGCAGACAAGCTGGAAAATTACAATGGATTGGCTGGTGCAATTGATAAGGTAGATGGAACCAATGTGTACTGGAAAGGAAATATTCACGCTAACGGGCGCCTTTACCTGACCACAAACGGTTTTGACTGTGGCCAGTATCAACAGTTCTTTTGTGGTGTCACTAATc                                                                                          >  1:162531/1‑301 (MQ=255)`  
                                                                                                                                                                                                                  `gTCAATACGCGACATCTGCTGGGAACTCTGCTTCCGCTGCGAATCAATCTGAGGTAAACGCTGAGAACTATGCCACAGCATCCGCTAACTATGCTCATTTGGAAGAACAGCAAGCAGACCGTGCGGAACGTGAGGCAGACAAGCTGGAAAATTACAATGGATTGGCTGGTGCAATTGATAAGGTAGATGGAACCAATGTGTACTGGAAAGGAAATATTCACGCTAACGGGCGCCTTTACATGACCACAAACGGTTTTGACTGTGGCCAGTATCAACAGTTCTTTGGTGGTGTCACTAATCg                                                                                         <  1:270182/301‑1 (MQ=255)`  
                                                                                                                                                                                                                  `atgtgtataagagacaggctgGGAACTCTGCTTCCGCTGCGCATCAATCTGAGGTAAACGCTGAGAACTCTGCCACAGCATCCGTTAACTATGCTCATTTGGCAGAACAGCAAGCAGACCGTGCGGAACGTGAGGCAGACAAGCTGGAAAATTACAATGGATTGGCTGGTGCAATTGATAAGGTAGATGGAACCAATGTGTACTGGAAAGGAAATATTCACGCTAACGGGCGCCTTTACCTGACCACAAACGGTTTTGACTGTGGCCAGTATCAACAGTTCTTTGGTGGTGTAACTAATCg                                                                                         <  1:7586/284‑1 (MQ=255)`  
                                                                                                                                                                                                                    `cAATACGCTACATCTGCTGGGAACTCTGCTTCCGCTGCGCATCAATCTGAGGTAAACGCTGAGAACTCTGCCACAGCATCCGCTAACTATGCTCATTTGGCAGAACAGCAAGCAGACCGTGCGGAACGTGAGGCAGACAAGCTGGAAAATTACAATGGATTGGCTGGTGCAATTGATAAGGTAGATGGAACCAATGTGTACTGGAAAGGAAATATTCACGCTAACGGGCGCCTTTACATGACCACAAACGGTTTTGACTGTGGCCAGTATCAACAGTTCTTTGGTGGTGTCACTAATCGtt                                                                                       >  1:443861/1‑301 (MQ=255)`  
                                                                                                                                                                                                                        `aCGCTACATCTGCTGGGAACTCTGCTTCCGCTGCGCATCAATCTGAGGTAAACGCTGAGAACTCTGCCACAGCATCCGCTAACTATGCTCATTTGGCAGAACAGCAAGCAGACCGTGCGGAACGTGAGGCAGACAAGCTGGAAAATTACAATGGATTGGCTGGTGCAATTGATAAGGTAGATGGAACCAATGTGTACTGGAAAGGAAATATTCACGCTAACGGGCGCCTTTACATGACCACAAACGGTTTTGACTGTGGCCAGTATCAACAGTTCTTTGGTGGTGTCACTAATCGTTActc                                                                                   <  1:202191/301‑1 (MQ=255)`  
                                                                                                                                                                                                                         `cGCTACATCTGCTGGGAACTCTGCTTCCGCTGCGCATCAATCTGAGGTAAACGCTGAGAACTCTGCCACAGCATCCGCTAACTATGCTCATTTGGCATAACAGCAAGCAGACCGTGCGGAACGTGAGGCAGACAAGCTGGAAAATTACCATGGATTGGCTGGTGCAATTGATAAGGTAGATGGAACAAATGTGTACTGGAAAGGAAATATTCACGCTAACGGGCGCCTTTACCTGAACACAAACTGGTTTGACTGTGGCCAGTATCAACAGTACTTTGGTGGTGTCACTactgactgttat                                                                                  >  1:251668/1‑290 (MQ=255)`  
                                                                                                                                                                                                                         `cGCTACATCTGCTGGGAACTCTGCTTCCGCTGCGCATCAATCTGAGGTAAACGCTGAGAACTCTGCCACAGCATCCGCTAACTATGCTCATTTGGCAGAACAGCAAGCAGACCGTGCGGAACGTGCGGCAGACAAGCTGGAAAATTACAATGGATTGGCTGGTGCAATTGATAAGGTAGATGGAACCAATGTGTACTGGAAAGGAAATATTCACGCTAACGGGCGCCTTTACATGACCACAAACGGTTTTGACTGTGGCCAGTATCAACAGTTCTTTGGTGGTGTCACTAATCGTTActct                                                                                  >  1:427227/1‑301 (MQ=255)`  
                                                                                                                                                                                                                         `cGCTACATCTGCTCGGAACTCTGCTTCCGCTGCGCATCAATCTGAGGCAAACGCTGAGAACTCTGCCACAGCATCCGCTAACTATGCTCATTTGGCAGAACAGCAAGCAGACCGTGCGGAACGTGAGGCAAACAAGCTGGAAAATTACACTGGATTGGCTGGTGCCATTGAAAAGGTAGATGGAACCAATGTGTACAGGAAAGGAAATATTCACGCTAACGAGCGCCTTTACATGACCACAAACGGTTTTGTCTGTGGACAGTATCAACAGTTCTTTGGTGGTGTCACTAATCGTTattat                                                                                  >  1:353638/1‑297 (MQ=255)`  
                                                                                                                                                                                                                                `atttctgGGAACTCTCCTACCGCTGCGCATCAATCGAAGGTAAACGCTGAGAACGCTGCCACAGCAGCCGCTAACTATGCTCATTTGGCAGAACAGCAAGCAGACCGTGCGGAACGTGAGGCAGACAAGCTGGAAAATTACAATGGATTGGCTGGTGCAATTGATAAGGTAGATGGAACCAATGTGTACTGGAAAGGAAATATTCACGCTAACGGGCGCCTTTACATGACCACAAACGGTTTTGACTGTGGCCAGTATCAACAGTTCTTTGGTTGTGTCACTAATCGTTACTCTGTCATgg                                                                           <  1:175155/297‑1 (MQ=255)`  
                                                                                                                                                                                                                                  `tgctgGGACCTCTGCATCCGCGGCGCATCAATCTGAGTGAAACGCTGAGAACGCTGCCACAGCACCCGCTAACTATGCTCATTTGGCAGAACAGCAAGCAGACCGTGCGGAACGTGAGGCAGACAAGCTTGAAAATTACAATGGATTGGCTGGTGCAATTGATAAGGTAGATGGAACCAATGTGTACTGGAAAGGAAATATTCACGCTAACGGGCGCCTTTACATGACCACAAACGGTTTTGACTGTGGCCAGTATCAACACTTCTTTGGTGGTGTCACTAATAGTTACTCTGTCATGGAg                                                                         <  1:226761/301‑1 (MQ=255)`  
                                                                                                                                                                                                                                  `tgctgGGAACTCTGCGTCCGCGGCGCATCAATGTGAGGTAAATGCTGAGAACTCTGCTACAGCATCAGCTAACTCTGCTCATTGGGCAGAACAGCGAGCAGACCGTGCGGAACGTGAGGCAGACAAGCTGGAAAATTACCATGGATTGGCTGGTGCAATTGATAAGGTAGATGGAACCGATGTGTACTGGAAAGGAAATATTCACGCTAACGGGCGCCTTTACATGACCACAAACGGTTTTGACTGTGGCCAGTATCAACAGTTCTTTGGTGGTGTCACTAATCGTTACTCTGTCATGGAg                                                                         <  1:362584/301‑1 (MQ=255)`  
                                                                                                                                                                                                                                   `gcttGGAACTCTGCTTCCGCTGCGCATCAATCTGAGGTAAACGCTGAGAACTCTGCCAAAGCATCCGCTAACTATGCTCATTTGGCAGAACAGCAAGCAGACCGTGCGGAACGTGAGGCAGACAAGCTGGAAAATTACAATGGATTGGCTGGTGCAATTGATAAGGTAGATGGAACCAATGTGTACTGGAAAGGAAATATTCACGCTAACGGGCGCCTTTACATGACCACAAACGGTTTTGACTGTGGCCAGTATCAACAGTTCTTTGGTGGTGTCACTAATCGTAACTCTGTCATGGAgg                                                                        >  1:423286/1‑300 (MQ=255)`  
                                                                                                                                                                                                                                   `gctgGGAACTCTGCTTCCGCTGCGCATCAATCTGAGGTAAACGCTGAGAACTCTGCCACAGCATCCGCTAACTATGCTCATTTGGCAGAACAGCAAGCAGACCGTGCGGAACGTGAGGCAGACAAGCTGGAAAATTACAATGGATTGGCTGGTGCAATTGATAAGGTAGATGGAACCAATGTGTACTGGAAAGGAAATATTCACGCTAACGGGCGCCTTTACATGACCACAAACGGTTTTGACTGTGGCCAGTATCAACAGTTCTTTGGTGGTGTCACTAATCGTTACTCTGTCATGGAgg                                                                        >  1:24040/1‑300 (MQ=255)`  
                                                                                                                                                                                                                                                 `ttCCGCTGCGCATCAATCTGAGGTAAACGCTGAGAACTCTGCCACAGCATCCGCTAACTCTGCTCATTTGGCAGAACAGCAAGCAGACCGTGCGGAACGTGAGGCAGACAAGCTGGAAAATTACAATGGATTGGCTGGTGCAATTGATAAGGTAGATGGAACCGATGTGTACTGGAAAGGAAATATTCACGCTAACGGGCGCCTTTACATGACCACAAACGGTTTTGACTGTGGCCAGTATCAACAGTTCTTTGGTGGTGTCACTAATCGTTACTCTGTCATGGAGTGGGGAGATGAGAAc                                                          <  1:463189/301‑1 (MQ=255)`  
                                                                                                                                                                                                                                                    `cGCTGCGCATCAATCTGGGGTAAACGCTGAGAACTATGCCACAGCATCCGCTAACTATGCTCATTTGGCAGAACAGCAAGCAGACCGTGCGGAACGTGAGGCAGACAAGCTGGAAAATTACAATGGATGGGCTGGTGCAATTGATAAGGTAGATGGAACCAATGTGTACTGGAAAGGAAATATTCACGCTAACGGGCGCCTTTACATGACCACAAACGGTTTTGACTGTGGCCAGTATCAACAGTTCTTTGGTGGTGTCACTAATCGTTACTCTGTCATGGAGTGGGGAGATGAGAACGGa                                                       <  1:5628/301‑1 (MQ=255)`  
                                                                                                                                                                                                                                                        `gcgcATCACTCTGAGGTAAACGCTGAGAACTATGCCACAGCATCCGCTAACTCTGCTCATTTGGCAAAACAGCAAGCAGACCGTGCGGAACGTGAGGCAGACAAGCTGGAAAATTACAATGGATTGGCTGGTGCAATTGATAAGGTAGATGGAACCAATGTGTACTGGAAAGGAAATATTCACGCTAACGGGCGCCTTTACATGACCACAAACGGTTTTGACTGTGGCCAGTATCAACAGTTCTTTGGTGGTGTCACTAATCGTTACTCTGTCATGGAGTGGGGAGATGAGAACGGATggg                                                   >  1:471862/1‑300 (MQ=255)`  
                                                                                                                                                                                                                                                             `tCAATCTGAGGTAAACGCTGAGAACTCTGCCACAGCATCCGCTAACTATGCTCATTTGGCAGAACAGCAAGCAGACCGTGCGGAACGTGAGGCAGACAAGCTGGAAAATTACAATGGATTGGCTGGTGCAATTGATCAGGTAGATGGAACCAATGTGTACTGGAAAGGAAATATTCACGCTAACGGGCGCCTTTACCTGACCACAAACGGTTTTGACTGTGGCCAGTATCAACAGTTCTTTGGTGGTGTCACTAATCGTTACTCTGTCATGGAGTGGGTATCTGAGAACGGATGGCTGatg                                              >  1:279696/1‑301 (MQ=255)`  
                                                                                                                                                                                                                                                               `aaTCTGAGGTAAACGCTGAGAACTCTGCCACAGCATCCGCTAACTATGCTCATTTGGCAGAACAGCAAGCAGACCGTGCGGAACGTGAGGCAGACAAGCTGGAAAATTACAATGGATTGGCTGGTGCAATTGATAAGGTAGATGGAACCAATGTGTACTGGAAAGGAAATATTCACGCTAACGGGCGCCTTTACATGACCACAAACGGTTTTGACTGTGGCCAGTATCAACAGTTCTTTGGTGGTGTCACTAATCGTTACTCTGTCATGGAGTGGGGAGATGAGAACGGATGGCTGatgta                                            <  1:106690/301‑1 (MQ=255)`  
                                                                                                                                                                                                                                                               `aaTCTGAGGTAAACGCTGAGAACTCTGCCACAGCATCCGCTAACTATGCTCATTTGGCAGAACAGCAAGCAGACCGTGCGGAACGTGAGGCAGACAAGCTGGAAAATTACAATGGATTGGCTGGTGCAATTGATAAGGTAGATGGAACCAATGTGTACTGGAAAGGAAATATTCAAGCTAACGGGCGCCTTTACATGACCACAAACGGTTTTGACTGTGGCCAGTATCAACAGTTCTTTGGTGGTGTCACTAATCGTTACTCTGTAATGGAGTGGGGAGATGAGAACGGATgctgtctctt                                            >  1:309547/1‑292 (MQ=255)`  
                                                                                                                                                                                                                                                                `aTCTGAGGTAAACGCTGAGAACTCTGCCACAGCATCCGCTAACTCTGCTCATTTGGCAGAACAGCAAGCAGACCGTGCGGAACGTGAGGCAGACAAGCTGGAAAATTACAATGGATTGGCTGGTGCAATTGATAAGGTAGATGGAACCAATGTGTACTGGAAAGGAAATATTCACGCTAACGGGCGCCTTTACATGACCACAAACGGTTTTGACTGTGGCCAGTATCAACAGTTCTTTGGTGGTGTCACTAATCGTTACTCTGTCATGGAGTGGGGAGATGAGAACGGATGGCTGatgtat                                           >  1:179341/1‑301 (MQ=255)`  
                                                                                                                                                                                                                                                                `aTCTGAGGTAAACGCTGAGAACTCTGCCACAGCATCCGCTAACTATGCTCATTTGGCAGAACAGCAAGCAGACCGTGCGGAACGTGAGGCAGACAAGCTGGAAAATTACAATGGATTGGCTGGTGCAATTGATAAGGTAGATGGAACCAATGTGTACTGGAAAGGAAATATTCACGCTAACGGGCGCCTTTACATGACCACAAACGGTTTTGACTGTCGCCAGTATACACAGTTCTTTGCTGGTGTCACTAATCGTTACTCTGTCATGGAGTGGGAAGATGCGCACGGATGGCTGatgtat                                           >  1:406557/1‑301 (MQ=255)`  
                                                                                                                                                                                                                                                                `aTCTGAGGTAAACGCTGAGAACTCTGCCACAGCATCCGCTAACTATGCTCATTTGGCAGAACAGCAAGCAGACCGTGCGGAACGTGAGGCAGACAAGCTGGAAAATGAAAAGGGATTGGCTGGTGCAATTGATAAGGTAGATGGAACCAATGTGTACTGGAAAGGAAATATTCAAGCTAACGGGCGCCTTTACCTGACCACAAACGGTTGTGACTGTGGCAAGTATCAACAGTTCTTTGGTGGTGTCACTAATGGTTACTCTGTCATGGAGTGGGTGGATGAGAACGGGTGGGTGatgtag                                           >  1:100/1‑300 (MQ=255)`  
                                                                                                                                                                                                                                                                `aTCTGAGGTAAACGCTGAGAACTCTGACACAGCATCCGCTAACTATGCTCATTTGGCAGAAAAGCAAGCAGACCGTGCGGAAAGAGAGGCAGACAAGCTGGAAAATTAAAATGGGTTGGCTGGTGAAATTGATAAGGTAGATGGAACCAATGAGTACTGGAAAGGAAATATTCACGCTAACGGGCGCCTTTAAATGACCACAAACGGTTTTGACTGTGGCCAGTATCAACAGTTATTTGGTGGTGTCACTAATCGTTACTCTGGCCTGGAGTGGGGCGATGAGAACGAATGGCTGatgtat                                           >  1:67103/1‑301 (MQ=255)`  
                                                                                                                                                                                                                                                                    `gAGGTCAACGCTGAGAACTCTGCCACAGCATCCGCTAACCATGCTCATTTGGCATAACAGCAAGCAGACCGGGCGGAACGTGAGGCAGACAAGCTGGAAAATTACAATGGATTGGCTGGTGCAATTGATAAGGTAGATGGAACCAATGTGTACTGGAAAGGAAATATTCACGCTAACGGTCGCCTTTACATGACCACAAACGGTTTTGACTGTGTCCAGTATCAACAGTTCTTTGGTGGTGTCACTAATCGTTACTCTGTCATGGAGTGGGGAGATGAGAACGGATGGCTGATGTATGTTc                                       <  1:218086/301‑1 (MQ=255)`  
                                                                                                                                                                                                                                                                    `gAGGTAAACGCTGAGAACTATGCCACAGCATCCGCTAACTATGCTCATTTGGCAGAACAGCAAGCAGACCGGGCGGAACGGGAGGCAGACAAGCTGGAAAATTACAATGGATTGGCTGGTGCAATTGATAAGGTAGATGGAACCAATGTGTACTGGAAAGGAAATATTCACGCTAACGGGCGCCTTTACATTACCACAAACGGTTTTGACTGTGGCCAGTATCAACAGTTCTTTGGTGGTGTCACTAATCGTTACTCTGTCATGGAGTGGGGAGATGAGAACGGATGGCTGATGTATGTTc                                       <  1:213714/301‑1 (MQ=255)`  
                                                                                                                                                                                                                                                                    `gAGGTAAACGCTGAGAACTATGACACAGCATCCGCTAAATATGCTCATTTGGCAGAACAGCAAGCAGACCGTGCGGAACGTGAGGGAGACAAGCTGGAAAATTACAATGGATTGGATGGTGCAATTGATAAGGTAGATGGAACCAATGTGTACTGGAAAGGAAATATTAACGCTAACGGGCGCCTTTACATGACCACAAACTGTTTTGACTGTGGCCAGTATCAACAGTTCTTTGGTGGTGTCACTAATCGTTACTCTGTCATGGAGTGGGGAGATGAGAACGGATGGCTGATGTATGTTc                                       <  1:418141/301‑1 (MQ=255)`  
                                                                                                                                                                                                                                                                           `aCGCTGAGAACTATGCCACAGCATACGCTAACTATGCTTATTTGGCCGAACAGCAAGCAGACCGTGCGGAACGTGAGGCAGACAAGCTGGAAAATTACAATTGAGTGGCTGGTTCAATTGATAAGGTCGATGGATCCAATGTTTACTGGAAAGGACATATTAACGCTACCGGTCGCCGTTACCTGACCACAAACTGTTTTGACTGTGGCCAGTATCAACAGTTCTTTGGTGGTGTCACTAATCGTTACTTTGTCATGGAGTGGGGAAATGAGAACGGATGGCTGATGTATGTTCAACGTag                                <  1:410266/301‑1 (MQ=255)`  
                                                                                                                                                                                                                                                                              `cTGAGAACTCTGCCACAGCATCCGCTAACTATGCTCATTTGGCAGAACAGCAAGCAGACCGTGCGGAACGTGAGGCAGACAAGCTGGAAAATTACAATGGATTGGCTGGTGCAATTGATAAGGTAGATGGAACCAATGTGTACTGGAAAGGAAATATTCACGCTAACGGGCGCCTTTACCTGACCACAAACGGTTTTGACTGAGGCCAGTATCAACAGTTCTTTGGTGGTGTCACTAATCGTTACTCTGTCATGGAGTGGGGAGATGAGAACGGATGGCTGCTGTATGTTCAACGTagaga                             >  1:120793/1‑301 (MQ=255)`  
                                                                                                                                                                                                                                                                                  `gaACTCTGCCACAGCATCCGCTAACTATGCTCATTTGGCAGAACAGCAAGCAGACCGTGCGGAACGTGAGGCAGACAAGCTGGAAAATTACAATGGATTGGCTGGTGCAATTGATAAGGTAGATGGAACCAATGTGTACTGGAAAGGAAATATTCACGCTAACGGGCGCCTTTACATGACCACAAACGGTTTTGACTGTGGCCAGTATCAACAGTTCTTTGGTGGTGTCACTAATCGTTACTCTGTCATGGAGTGGGGAGATGAGAACGGATGGCTGATGTATGTTCAACGTAGAGAGTgg                         >  1:452674/1‑301 (MQ=255)`  
                                                                                                                                                                                                                                                                                     `ctctGCCACAGCATCCGCTAACTCTGCTCATTTGGCAGAACAGCAAGCAGACCGTGCGGAACGTGAGGCAGACAAGCTGGAAAATTACAATGGATTGGCTGGTGCAATTGATAAGGTAGATGGAACCAATGTGTACTGGAAAGGAAATATTCACGCTAACGGGCGCCTTTACATGACCACAAACGGTTTTGACTGTGGCCAGTATCAACAGTTCTTTGGTGGTGTCACTAATCGTTACTCTGTCATGGAGTGGGGAGATGAGAACGGATGGCTGATGTATGTTCAACGTAGAGAGTGGaca                      <  1:134431/301‑1 (MQ=255)`  
                                                                                                                                                                                                                                                                                       `atGCCACAGCATCCGCTAACTATGCTCATTTGGCAGAACAGCAAGCAGACCGTGCGGAACGTGAGGCAGACAAGCTGGAAAATTACAATGGATTGGCTGGTGCAATTGATAAGGTAGATGGAACCAATGTGTACTGGAAAGGAAATATTCACGCTGACGGGCGCCTTTACATGACCACAAACGGTTTTGACTGTGGCCAGTATCAACAGTTCTTTGGTGGTGTCACTAATCGTTACTCTGTCATGGAGTGGGGAGATGAGAACGGATGGCTGATGTATGTTCAACGTAGAGAGTGGacaaa                    >  1:138694/2‑300 (MQ=255)`  
                                                                                                                                                                                                                                                                                                  `tCCGCTAACTATGCTCATTTGGCAGAACAGCAAGCAGACCGTGCGGAACGTGAGGCAGACAAGCTGGAAAATTACAATGGATTGGCTGGTGCAATTGATAAGGTAGATGGAACCGATGTGTACTGGAAAGGAAATATTCACGCTAACGGGCGCCTTTACATGACCACAAACGGTTTTGACTGTGGCCAGTATCAACAGTTCTTTGGTGGTGTCACTAATCGTTACTCTGTCATGGAGTGGGGAGATGAGAACGGATGGCTGATGTATGTTCAACGTTGAGAGTGGACAACAGCGATCGGCg         >  1:116501/1‑301 (MQ=255)`  
                                                                                                                                                                                                                                                                                                  `tCCGCTAACTATGCTCATTTGGCAGAACAGCAAGCAGACCGTGCGGAACGTGAGGCAGACAAGCTGGAAAATTACAATGGATTGGCTGGTGCAATTGATAAGGTAGATGGAACCAATGTGTACTGGAAAGGAAATATTCACGCTAACGGGCGCCTTTACCTGACCACAAACGGTTTTGACTGTGGCCAGTATAAACAGTTCTTTGGTGGTGTCACTAATCGTTACTATGTCATGGAGTGGGGAGATGAGAACGGATGGCTGATGTATGTTCAACGTAGAGAGTGGACAACAGCGATAGGCg         >  1:165833/1‑301 (MQ=255)`  
                                                                                                                                                                                                                                                                                                         `aCTCTGCTCATTTGGCAGAACAGCAAGCAGACCGTGCGGAACGTGAGGCAGACAAGCTGGAAAATTACAATGGATTGGCTGGTGCAATTGATAAGGTAGATGGAACCGATGTGTACTGGAAAGGAAATATTCACGCTAACGGGCGCCTTTACATGCCCACAAACGGTTTTGACTGTGGCCAGTATCAACAGTTCTTTGGTGGTGTCACTAATCGTTACTCTGTCATGGAGTGGGGAGATGAGAACGGATGGCTGATGTATGTTCAACGTAGAGAGTGGacaactctctacgttgaacatac  >  1:197173/1‑283 (MQ=255)`  

                                                                                                                                                                                                                                                                                                            `|`                                                                                                                                                                                                                                                                                                           
`CTGTTCCGTTTGGTCAACTAAAGACCATGAACCAGAACTCATGGCAAGCACGTAATGAAGCCTTACAGTTCCGTAATGAGGCTGAGACTTTCAGAAACCAAGCGGAGGGCTTTAAGAACGAGTCCAGTACCAACGCTACGAACACAAAGCAGTGGCGCGATGAGACCAAGGGTTTCCGAGACGAAGCCAAGCGGTTCAAGAATACGGCTGGTCAATACGCTACATCTGCTGGGAACTCTGCTTCCGCTGCGCATCAATCTGAGGTAAACGCTGAGAACTCTGCCACAGCATCCGCTAACTCTGCTCATTTGGCAGAACAGCAAGCAGACCGTGCGGAACGTGAGGCAGACAAGCTGGAAAATTACAATGGATTGGCTGGTGCAATTGATAAGGTAGATGGAACCAATGTGTACTGGAAAGGAAATATTCACGCTAACGGGCGCCTTTACATGACCACAAACGGTTTTGACTGTGGCCAGTATCAACAGTTCTTTGGTGGTGTCACTAATCGTTACTCTGTCATGGAGTGGGGAGATGAGAACGGATGGCTGATGTATGTTCAACGTAGAGAGTGGACAACAGCGATAGGCGGTAACAT  >  NC_001604.1/35066‑35663`

  

Alignment Legend

Aligned base mismatch/match (shaded by quality score): `ATCG/ATCG` `< 3 ≤` `ATCG/ATCG` `< 9 ≤` `ATCG/ATCG` `< 19 ≤` `ATCG/ATCG` `< 33 ≤` `ATCG/ATCG` `< 38 ≤` `ATCG/ATCG`

Unaligned base: `atcg`    Masked matching base: `atcg`    Alignment gap: `‑`    Deleted base: `‑`
